# Master microRNA-222 regulates cardiac microRNA maturation and triggers Tetralogy of Fallot

**DOI:** 10.1038/s41392-022-00993-1

**Published:** 2022-05-30

**Authors:** Chao Li, Hongdou Li, Xiaoying Yao, Dong Liu, Yongming Wang, Xinyi Huang, Zhongzhou Yang, Wufan Tao, Jian-Yuan Zhao, Hongyan Wang

**Affiliations:** 1 Obstetrics and Gynecology Hospital, NHC Key Laboratory of Reproduction Regulation, Shanghai Institute of Planned Parenthood Research, State Key Laboratory of Genetic Engineering, School of Life Sciences, and Children’s Hospital, Fudan University, 200438 Shanghai, China; 2 Shanghai Key Laboratory of Metabolic Remodeling and Health, Institute of Metabolism and Integrative Biology, Institute of Reproduction and Development, Fudan University, 200438 Shanghai, China; 3 School of Life Science, Key Laboratory of Neuroregeneration of Jiangsu and Ministry of Education, Co-innovation Center of Neuroregeneration, Nantong University, 226019 Nantong, China; 4 State Key Laboratory of Pharmaceutical Biotechnology and MOE Key Laboratory of Model Animal for Disease Study, Nanjing University Medical School, 211166 Nanjing, China; 5 Institute of Developmental Biology & Molecular Medicine, Fudan University, 200438 Shanghai, China

**Keywords:** Disease model, Non-coding RNAs

**Dear Editor**,

Tetralogy of Fallot (TOF) is the most common complex congenital heart disease. Besides gene mutations and copy number variants, altered protein function induced by post-transcriptional or translational regulation also contributes to the onset of TOF.^1^ MiRNAs are short noncoding RNAs that bind to the 3’-UTR of target mRNAs to repress protein production. However, the causal link between miRNAs and TOF and the underlying mechanism has not been established.

To identify TOF-related miRNAs, we used a data mining approach to detect differentially expressed miRNAs in the myocardium of patients with TOF from the USA^2^ and China.^3^ Five miRNAs with upregulated expression were identified in both TOF datasets (Fig. 1a) and validated in five pairs of right ventricle tissues from aborted fetuses with TOF and control. We found that only the expression levels of miR-222 and miR-187 showed significantly higher expression in cases with TOF than in controls (Fig. 1b and Supplementary Fig. S1a). MiR-222 was the most abundant miRNA among all upregulated miRNAs (Fig. 1c) and had a higher percentage in TOF than that in controls (Fig. 1d); these results suggested that increased cardiac miR-222 levels in human embryos might contribute to the onset of TOF.

**Fig. 1:**
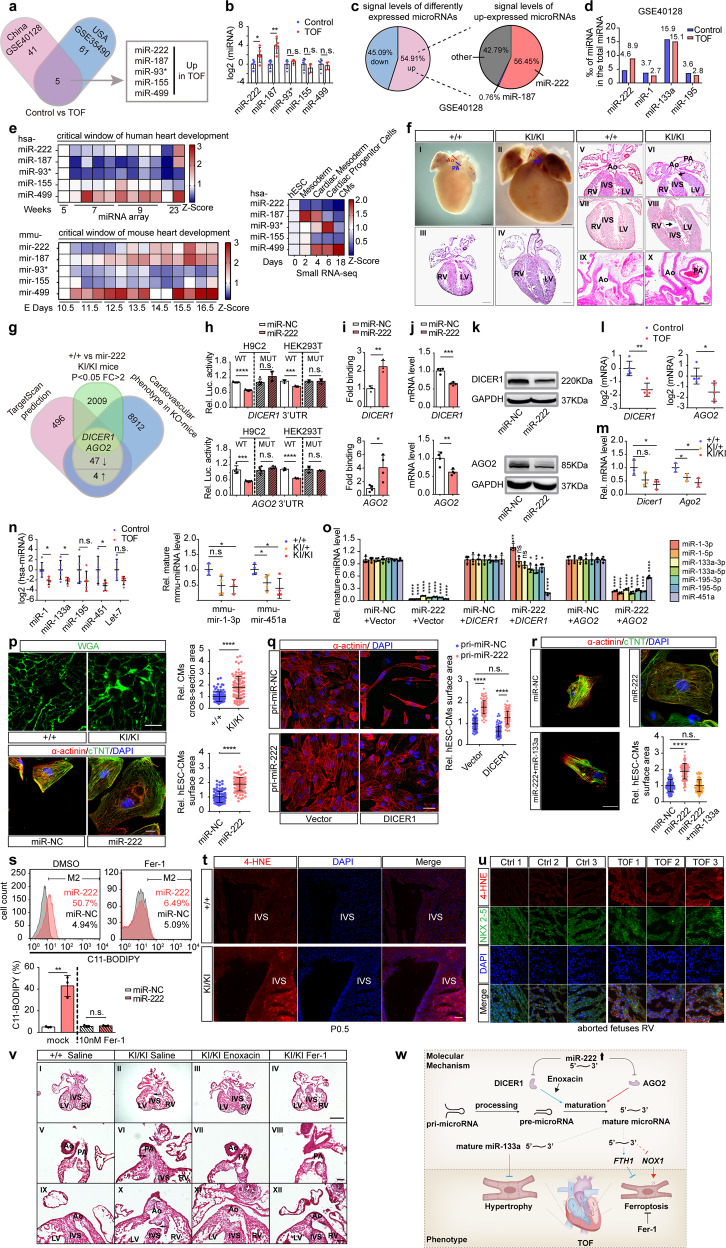
Master microRNA-222 regulates cardiac microRNA maturation and triggers Tetralogy of Fallot. **a** Schematic illustration of the screening process for differentially expressed microRNAs (fold change >2, *P* < 0.05) using two independent datasets (GSE35490 and GSE40128). **b** RT-qPCR analysis of miR-222 levels in the RVs of aborted fetuses with TOF (*n* = 5) and normal controls (*n* = 5). **c** Abundance of upregulated microRNAs (fold change >2, *P* < 0.05) in TOF samples vs. normal using the GSE40128 dataset. **d** Abundance of miR-222, miR-1, miR-133a, and miR-195 in TOF samples vs. normal using the GSE40128 dataset. Abundance was calculated by the probe signal levels of indicated miRNA divided by total miRNA. **e** Temporal analysis of miR-222 expression during normal human heart development by microarray analyses (upper, left) and mouse heart development using the GSE105834, GSE82960, GSE105910, GSE82604, GSE82822, GSE82942, and GSE101175 datasets (lower, left) and differentiation of hESCs into CMs by RNA-seq (right). **f** (I–X) Stereoscopic images of whole hearts (I, II) and H&E-stained heart sections (III–X) from homozygous mir-222 KI and control mice, displaying human TOF-like phenotypes, such as (I) the control heart shows a normal caliber ratio of the pulmonary artery (PA) and aorta (Ao); (II) A mir-222 KI littermate of the animal in (I) shows narrowing of the pulmonary artery, RV hypertrophy (IV), overriding aorta (arrow, VI), VSDs (arrow VIII), and small aortic root (star, X) at P0.5; The scale bars in (I–IV) and (V–X) are 500 µm and 200 µm, respectively. **g** Schematic illustration of the screening approach for target genes of miR-222 using TargetScan prediction, RNA-seq, MGI database, and RT-qPCR verification. **h** Luciferase assays of H9C2 or HEK293T cells cotransfected with miR-222 or scramble control and luciferase reporter plasmids containing WT or mutant *DICER1* 3′UTR (upper) and *AGO2* (lower) 3′UTR. **i** Biotin-labeled miR-222 and scramble control were transfected into hESC-CMs and the human *DICER1* (upper, *n* = 3) or *AGO2* (lower, *n* = 4) 3′-UTR pulled down by miR-222 or scramble control was quantified by RT-qPCR. **j**, **k** RT-qPCR and western blotting analysis of mRNA (**j**) and protein (**k**) levels, respectively, of *DICER1* (upper) and *AGO2* (lower) in hESC-CMs transfected with miR-222 or scramble control. **l** RT-qPCR analysis of the mRNA levels of *DICER1* (left) and *AGO2* (right) in the RVs of aborted fetuses with TOF and control fetuses (*n* = 5). **m** RT-qPCR analysis of the mRNA levels of *Dicer1* and *Ago2* in hearts of P0.5 neonatal mice of the indicated genotypes (*n* = 3). **n** RT-qPCR analysis of the mature miRNA levels of miR-1, miR-133a, miR-195, miR-451a, and Let-7 in the RVs of aborted fetuses with TOF and control fetuses (*n* = 5) (left); RT-qPCR analysis of mature mmu-mir-1 and mmu-mir-451a levels in hearts of P0.5 neonatal mice of the indicated genotypes (*n* = 3) (right). **o** RT-qPCR analysis of the levels of the mature forms of DICER1-dependent miRNAs (miR-1, miR-133a, miR-195) and AGO2-dependent miRNA (miR-451a) in hESC-CMs transfected with miR-222 or scramble control only (left) or cotransfected with miRNA and the indicated expression plasmids (middle and right) for 72 h (*n* = 4). **p** Immunofluorescence staining of WGA in heart sections from P0.5 neonatal mice and quantification of the cross-section area of CMs (upper). Immunofluorescence staining of the hESC-CMs markers α-actinin (red) and cTnT (green) in miR-222- or scramble control-transfected hESC-CMs and quantification of the surface area of hESC-CMs (lower). **q** Immunofluorescence staining (left) of α-actinin (red) in hESC-CMs expressing exogenous pri-miR-222 and transfected with the indicated genes and quantification (right) of the surface area of CMs. **r** Immunofluorescence staining of the hESC-CMs markers α-actinin (red) and cTnT (green) in miR-222- only, miR-222- and miR-133a- or scramble control-transfected hESC-CMs and quantification of the surface area of hESC-CMs; at least 100 cells were quantified in each group. The scale bars in (**p**, upper), (**q**, left), and (**p**, lower, **r**) represent 20 µm, 10 µm, and 50 µm, respectively. **s** FACS analysis and quantification of lipid ROS levels (C11-BODIPY) in hESC-CMs transfected with miR-222 or scramble control by Lipofectamine RNAimax with or without Fer-1 treatment. **t** Representative immunofluorescence staining of the lipid ROS marker 4-HNE in heart sections from P0.5 mice and normal controls with the indicated genotypes. **u** Representative immunofluorescence staining of NKX2-5 and the lipid ROS marker 4-HNE in heart sections from RV tissues from aborted fetuses with TOF (*n* = 3) and normal controls (*n* = 3). **v** H&E-stained heart sections from control mice and homozygous mir-222 KI treated with saline, enoxacin, or Fer-1 as indicated, which displayed human TOF-like phenotypes, such as pulmonary stenosis (VI), overriding aorta (arrow, X), and VSDs (arrow in II) at E13.5. The scale bars in (I–IV) and (V–XII) represent 500 µm and 100 µm, respectively. **w** Schematic diagram of the role of the miR-222-*DICER1*-*AGO2* axis in the pathogenesis of TOF. miR-222: has-miR-222-3p (MI0000299); miR-187: has-miR-187-3p (MI0000274); miR-93*: has-miR-93-3p (MI0000095); miR-155: has-miR-155-5p (MI0000681); miR-499: has-miR-499-5p (MI0003183); miR-1: has-miR-1-3p; miR-133a: has-miR-133a-3p; miR-195: has-miR-195-5p; miR-451a: has-miR-451; mir-222: mmu-mir-222-3p; CMs: cardiomyocytes; +/+: WT; + /KI: heterozygous H11-Myh7-mir-222 knock-in, KI/KI: homozygous H11-Myh7-mir-222 knock-in; Ao: aorta; PA: pulmonary artery; RV: right ventricle; LV: left ventricle; IVS: interventricular septum; Rel.: relative; Luc.: luciferase; Ctrl: control. For all immunofluorescence staining experiments, DAPI was used for nuclear staining (blue). U6 or GAPDH was used as an internal control. Data are shown as means ± SD. *P < 0.05, **P < 0.01, ***P < 0.001, ****P < 0.0001

We next examined the physiological levels of miR-222 during normal embryonic development in vivo and cardiomyocyte differentiation in vitro. During two to eight weeks of human embryonic heart development, miR-222 levels decreased from week 5 to week 7 and then gradually increased based on our microarray analysis; In mouse, mmu-mir-222 have a similar expression pattern in the developing heart.^4^ Furthermore, miR-222 expression gradually decreased during the differentiation of human embryonic stem cells (hESCs) to human embryonic stem cell-derived cardiomyocytes (hESC-CMs) based on our previous data (Fig. 1e).^5^ These findings suggest that a gradual decrease in miR-222 expression is a prerequisite for proper early embryonic heart development, abnormally high miR-222 levels might disrupt this process.

To assess the consequences of high miR-222 expression in the developing heart, we generated mir-222 knock-in (KI) mice in which the expression of exogenous mmu-mir-222 was under the control of the β-myosin heavy chain (β-MHC) promoter (Supplementary Fig. S2a). The β-MHC promoter drives the expression of cardiomyocyte-specific genes from E9.5. Levels of mmu-mir-222 in the heart were approximately sixfold higher in homozygous KI mice than in wild-type (WT) (Supplementary Fig. S2b). Interbreeding of heterozygotes yielded the Mendelian ratio (24:77:39) of KI/KI, KI/ + , and + /+ offspring, indicating embryonic lethality caused by the homozygotes mir-222 KI. The body weights did not differ significantly among the groups; however, the ratio of heart weight to body weight was dramatically higher in KI/KI mice than in WT mice (Supplementary Fig. S2c). Approximately 20.8% (5/24) of KI/KI mouse pups died immediately after birth due to asphyxia, and 54.2% (13/24) of neonatal KI/KI mice had at least one cardiac defect. Typical features of human TOF were observed separately or in combination in the hearts of KI/KI mice (Fig. 1f and Supplementary Fig. S2d). Together with the observation that the cardiac defect frequencies in KI/ + mice are lower than KI/KI mice but higher than WT mice (Supplementary Fig. S2e, S2f), these results demonstrate that embryonic cardiomyocyte-specific expression of exogenous mmu-mir-222 in mice can recapitulate the phenotypes of human TOF.

To identify miR-222 targets involved in the pathogenesis of TOF, we predicted 82 miR-222 target genes based on the TargetScan7.1 database (http://www.targetscan.org) and TOF-related genes in the MGI mouse phenotype database. Among the 82 candidates, we verified 47 downregulated genes and 4 upregulated genes using RT-qPCR (Supplementary Fig. S3a). The downregulated genes *DICER1* and *AGO2* were finally selected for further investigation, as they play crucial roles in heart development and function coordinately in the process of miRNA biogenesis to negatively regulate gene expression (Fig. 1g), in accord with the top two significantly enriched Gene Ontology terms in RNA-seq results (Supplementary Fig. S3b). To confirm, we demonstrated that miR-222 binds directly to the 3′UTRs of *DICER1* and *AGO2* by luciferase reporter assays and miRNA RNA-IP assays (Fig. 1h, i and Supplementary Fig. S3c, S3d). In vitro, exogenous miR-222 reduced the expression of endogenous *DICER1* and *AGO2* at both the mRNA and protein levels in hESC-CMs and hESCs (Fig. 1j, k and Supplementary Fig. S3e). In vivo*, DICER1*/*AGO2* and *Dicer1*/*Ago2* mRNA levels were significantly lower in the RVs of fetuses with TOF and mir-222-KI mice compared to controls (Fig. 1l, m and Supplementary Fig. S3f, S3g). These findings indicated that upregulated miR-222 simultaneously downregulates the expression of *DICER1* and *AGO2*, both of which are specifically responsible for processing from pre-miRNA to mature miRNA. We then tested whether the upregulation of miR-222 expression impairs the biogenesis of miRNAs. Indeed, the levels of mature cardiac-related miRNAs were decreased in fetuses with TOF and mir-222-KI mice at P0.5 compared to controls (Fig. 1n). Furthermore, miR-222-induced decreasing levels of the mature forms of cardiac-related miRNAs can be specifically reversed by the coexpression of *DICER1* or *AGO2* in hESC-CMs (Fig. 1o and Supplementary Fig. S3h, S3i). Particularly, our finding that mature miR-222 levels are regulated by *DICER1* (Supplementary Fig. S3k), suggested a possible feedback regulation system to maintain a low level of miR-222 in a physiological state. Taken together, our data suggest that miR-222 inhibits cardiac miRNA maturation by simultaneously downregulating the expression of both *DICER1* and *AGO2*, which consequently contributes to TOF.

Cardiomyocyte hypertrophy is a typical phenotype of human TOF. MiR-222 upregulation increased the cardiomyocyte cross-section area in KI/KI mice and hESC-CMs (Fig. 1p and Supplementary Fig. S4a). Markers of cardiomyocyte hypertrophy increased in response to high levels of miR-222 expression in both fetuses with TOF and in hESC-CMs (Supplementary Fig. S4b–d). MiR-133a, a key regulator of cardiac hypertrophy, was inhibited by the miR-222/*DICER1* axis (Fig. 1o), and exogenous miR-222-induced hypertrophy in hESC-CMs can be nearly reversed by exogenous *DICER1* (Fig. 1q) or miR-133a (Fig. 1r). These results demonstrate that the upregulation of miR-222 expression induces cardiomyocyte hypertrophy at least in part by inhibiting *DICER1*-miR-133a signaling.

Besides hypertrophy, exogenous miR-222 significantly increased levels of lipid peroxides in hESC-CMs, which could be abrogated by the ferroptosis inhibitor ferrostatin-1 (Fer-1) (Fig. 1s and Supplementary Fig. S5a). Similarly, in CMs from neonatal KI/KI mice, lipid peroxide levels were nearly twofold higher, and the levels of 4-hydroxynonenal (4-HNE), a product of lipid peroxidation, were significantly increased in the heart, especially in the interventricular septum, compared with levels in matched controls (Fig. 1t and Supplementary Fig. S5b). Importantly, 4-HNE levels in heart tissues from fetuses with TOF were also higher than those in controls (Fig. 1u). These results collectively suggested that miR-222 upregulation induced ferroptosis in vitro and in vivo. We further demonstrated that ferroptosis induced by miR-222 was mediated by the downregulated *DICER1* and *AGO2*, as exogenous *DICER1* or *AGO2* can fully reverse the miR-222-induced increase in lipid peroxidation levels (Supplementary Fig. S5e).

Finally, we examined whether mir-222-induced cardiac defects could be reversed by the miRNA maturation enhancer enoxacin or ferroptosis inhibitor Fer-1 in KI/KI mice. Our results revealed that both enoxacin and Fer-1 treatments significantly reduced embryonic heart defects, respectively, including phenotypes of VSD, subpulmonary stenosis, and overriding aorta (Fig. 1v and Supplementary Fig. S6). Overall, these in vivo data showed that enoxacin and Fer-1 effectively prevent mir-222-induced TOF-related cardiac defects.

In summary, we identified miR-222 as a master regulator of cardiac miRNA maturation/dosage and demonstrated connections among high levels of miR-222, cardiac hypertrophy, ferroptosis in mouse models and fetuses with TOF (Fig. 1w). Although the triggers of upregulated miR-222 remain further studied, our study shed light on the role of miR-222-*DICER1*-*AGO2* signaling in the control of cardiac miRNA maturation/dosage and ferroptosis as potential therapeutic targets for the prevention of TOF.

## Supplementary information

Supplementary Materials

Supplemental Excel File I

Supplemental Excel File II

Supplemental Excel File III

## Data Availability

The data are available from the corresponding author on reasonable request.

## References

[1] Porrello ER (2013). microRNAs in cardiac development and regeneration. Clin. Sci..

[2] O’Brien JE (2012). Noncoding RNA expression in myocardium from infants with Tetralogy of Fallot. Circ. Cardiovasc. Genet..

[3] Zhang J (2013). MicroRNA deregulation in right ventricular outflow tract myocardium in nonsyndromic Tetralogy of Fallot. Can. J. Cardiol..

[4] Rahmanian S (2019). Dynamics of microRNA expression during mouse prenatal development. Genome Res..

[5] Xie Y (2020). MircroRNA-10b promotes human embryonic stem cell-derived cardiomyocyte proliferation via novel target gene LATS1. Mol. Ther. Nucleic Acids.

