# Supplementary material for: Master microRNA-222 regulates cardiac microRNA maturation and triggers Tetralogy of Fallot
Source: Signal Transduct Target Ther. 2022 May 30;7:165. doi: 10.1038/s41392-022-00993-1 (PMC9148908; doi:10.1038/s41392-022-00993-1)
Supplement: Supplementary file 1 — Supplementary Materials [file 41392_2022_993_MOESM1_ESM.pdf]

Supplementary Materials for

Master microRNA-222 regulates cardiac microRNA maturation and triggers tetralogy of Fallot

Chao Li [1,2,†]· Hongdou Li [1,2,†]· Xiaoying Yao[1]· Dong Liu [5]· Yongming Wang[1]· Xinyi Huang[4]· Zhongzhou Yang[4]· Wufan Tao[3]· Jian-Yuan Zhao[1,*]· Hongyan Wang [1,2,*]

Correspondence to: wanghy@fudan.edu.cn, zhaojy@fudan.edu.cn

[1] Obstetrics and Gynecology Hospital, NHC Key Laboratory of Reproduction Regulation, Shanghai Institute of Planned Parenthood Research, State Key Laboratory of Genetic Engineering at School of Life Sciences, Children's Hospital, Fudan University, Shanghai 200438, China.

[2] Shanghai Key Laboratory of Metabolic Remodeling and Health, Institute of Metabolism and Integrative Biology, Institute of Reproduction and Development, Fudan University, Shanghai 200438, China.

[3] Institute of Developmental Biology & Molecular Medicine, Fudan University, Shanghai 200438, China.

[4] State Key Laboratory of Pharmaceutical Biotechnology and MOE Key Laboratory of Model Animal for Disease Study, Nanjing University Medical School, Nanjing 211166, China.

[5] School of Life Science, Key Laboratory of Neuroregeneration of Jiangsu and Ministry of Education, Co-innovation Center of Neuroregeneration, Nantong University, Nantong 226019, China.

† Chao Li and Hongdou Li contributed equally to this work.

This PDF file includes:

Materials and Methods
Extended results and discussion
Supplementary Text
Figures S1 to S6
Tables S1 to S4
Figure S1 MiR-222 promotes cardiomyocyte differentiation
Figure S2 Cardiac-specific expression of exogenous miR-222 drives TOF-related phenotypes
Figure S3 MiR-222 disturbs miRNA maturation
Figure S4 MiR-222 induces cardiomyocyte hypertrophy
Figure S5 MiR-222 induces ferroptosis in CMs
Figure S6 MiR-222-induced cardiac defects could be reversed by enoxacin or Fer-1
Table S1 Gestational age and Sex of subjects.
Table S2 MiRNA expression in normal human heart development by microarray.
Table S3 Filtering with the MGI mouse phenotype database
Table S4. List of primer, mimic and inhibitor sequences used in this study

Materials and methods

Human tissue samples

All experiments involving human tissue samples and animals were conducted with approval from the Medical Ethics Committee at the Obstetrics & Gynecology Hospital of Fudan University. All experiments involving human tissue samples were performed in accordance with the Declaration of Helsinki. Clinical miscarried fetal samples were obtained from the Obstetrics & Gynecology Hospital of Fudan University with parental informed consent. Right ventricular myocardial, brain, liver, lung, and kidney tissues were surgically excised from ~20-week miscarried fetuses with TOF. All fetuses with nonsyndromic TOF (n = 5) were diagnosed by echocardiography in obstetrics clinics. Sex- and age-matched miscarried fetuses without TOF were used as controls (n = 5).

Mouse studies

All experimental procedures were carried out in accordance with the Administrative Panel on Laboratory Animal Care protocol and the institutional guidelines by Medical Ethics Committee at the Obstetrics & Gynecology Hospital of Fudan University. Hipp11 (H11) site-specific mir-222-knockin mice (hereafter called mir-222-KI mice), in which the expression of a single copy of exogenous mmu-mir-222 was under the control of the mouse β-MHC promoter, were generated using the CRISPR/Cas9 system on a C57BL/6J background.

Preparation of mRNA and DNA for Microinjection. Capped mRNAs for Cas9 was generated by using a mMESSAGEmMACHINE in vitro transcription kit (Invitrogen, AM1344) according to the manufacturer's instructions. The integrity of the RNA was assessed by electrophoresis on a 1% agarose gel. Before loading on the gel, the RNA was denatured by using the loading buffer provided in the Invitrogen kit according to the manufacturer's instructions. Plasmid DNA was prepared using a standard procedure and was subsequently extracted with phenol/chloroform. The DNA was diluted to 10 ng/μL by sterile microinjection TE buffer (0.1 mM EDTA, 10 mM Tris, pH 7.5) and was kept at −80 °C until the injection. The DNA was tested to be RNase-free by incubation with an in vitro transcribed RNA at 37 °C for 1 h and then by analyzing the mix on a 1% agarose gel. Before loading on the gel, the RNA was denatured as described above.

Microinjection for Generation of Hipp11 (H11) site-specific mir-222-knockin mice. A donor plasmid containing an expression cassette, in which a 661 bp mouse miR-222 genomic fragment including mmu-miR-222 sequence (5'- CCCTCAGTGGCTCAGTAGCCAGTGTAGATCCTGTC TTTGGTAATCAGCAGCTACATCTGGCTACTGGGTCTCTGGTGGC- 3') and flanking region was controlled by a 6.4 kb β-myosin heavy chain promoter[1] and a rabbit globin polyA signal sequence, was first constructed. Then, the donor vector, Cas9 mRNA, and single guide RNA (sgRNA, 5' -CTGAGCCAACAGTGGTAGTA- 3') targeting the H11 locus were co-microinjected into fertilized eggs from C57BL/6J mice, and transferred to pseudopregnant mice. After confirmation of stable Mendelian transmission, cardiac-specific mmu-mir-222 expression was confirmed by RT-qPCR. All injection mixes contained 5 ng/μL DNA and 50 ng/μL of in vitro transcribed Cas9 mRNA in microinjection TE buffer (0.1 mM EDTA, 10 mM Tris, pH 7.5). The injection mixes were prepared fresh before each injection by mixing equal volumes of 10 ng/μL DNA solution and 100 ng/μL mRNA solution.

To test animals for site-specific insertions, we performed three PCRs: one for the 5′-end junction, one for the 3′-end junction, and one internal to the transgene. The genomic DNA of their progeny was subjected to the verification of positive homologous recombination by PCR. Homozygous miR-222-KI mice were established by backcrossing the established mice with WT C57BL/6J mice, which was followed by heterozygous self-crossing. These lines were maintained by intercrosses between homozygous animals and they were fertile.

Hearts of P0 neonatal KI/KI mice (n = 3) and WT control mice (n = 3) were collected for an RNA-seq assay performed by BGI Genomics (Shenzhen, China).

For *in vivo* drug treatment, Fer-1 (Selleck, Houston, TX, USA; S7243) was dissolved in DMSO and then diluted in sterile saline and enoxacin (Sigma-Aldrich, St. Louis, MO, USA; 557305) was dissolved in sterile saline.Pregnant female mice were given a daily intraperitoneal injection of Fer-1 (1 mg/kg), enoxacin (15 mg/kg), or an equivalent volume of saline from E0.5 to E12.5. Embryonic hearts (E13.5) were collected for a histological analysis.

<u>Cell culture</u>

Human embryonic kidney 293T (HEK293T) cells and rat cardiac myocytes (H9C2 cells) were cultured in DMEM (Gibco, Waltham, MA, USA; 11995073) with 10% fetal bovine serum (Corning,

Inc., Corning, NY, USA; 35076111) and 1‰ Plasmocin (InvivoGen, San Diego, CA, USA; ant-mpt) at 37°C and 5% CO_2.

The H9 (WA09) human embryonic stem cell line (obtained from WiCell Research Institute, Madison, WI, USA) was cultured in mTeSR1 medium (StemCell Technologies, Vancouver, Canada, 85850) on Matrigel-coated 6-well plates (Corning) at 37°C under 5% CO_2. The cells were seeded at a density of 5×10^5 cells/well, and the medium was replaced every day.

Cardiac differentiation of hESCs

hESCs were differentiated into hESC-CMs by manipulating the Wnt signaling pathway as described previously[2], with modifications. Briefly, when hESCs cultured on Matrigel in mTeSR1 reached 90% confluence, the cells were treated with 10 μM CHIR-99021 (Selleck, Houston, TX, USA; S1263) in RPMI 1640/B27 supplement minus insulin (Gibco; A1895601) for one day (day 0 to day 1). The medium was changed to RPMI 1640/B27 supplement minus insulin, and the cells were cultured for 2 days. Then, the cells were cultured in the same medium with 5 μM IWR-1 (Sigma-Aldrich; I0161) for another 2 days. On day 7, the medium was replaced with RPMI 1640/B27 supplement with insulin (Gibco; 17504044) and cultured for 2 days again. On day 9, cardiomyocyte beating was observed under an inverted microscope. hESC-CMs were passaged using a CardioEasy CM Dissociation Enzyme Set (Cellapy, Beijing, China; CA2011100 and CA2012100) and seeded at a density of 5×10^6 cells/well in Matrigel-coated 6-well plates (Corning; 354277, 1:200). The medium was changed every 2 days until hESC-CMs recovered beating. Then, the medium was replaced with CardioEasy human cardiomyocyte purification medium (Cellapy; CA2005100), and the cells were cultured for 3 days. Next, the hESC-CMs were subjected to normal maintenance in RPMI 1640/B27 supplement plus insulin, with medium replacement being performed every 2 days.

Transfection

The miRNA mimic (B02004) and miRNA inhibitor (B03004) were purchased from GenePharma (Shanghai, China). Before transfection, hESC-CMs were purified 1 or 2 times until the purity of the cardiomyocytes reached at least 90%. The purified hESC-CMs were transfected with the miRNA mimic, miRNA inhibitor, siRNA, or respective negative control (NC) with

Lipofectamine RNAiMAX (Invitrogen, Carlsbad, CA, USA; 13778075) or cotransfected with the miR-222 mimic and *DICER1*- or *AGO2*-expressing plasmids with Lipofectamine 3000 (Invitrogen; L3000015). Cells were harvested 48 h after transfection.

Histological analysis

Hearts at E13.5 or P0.5 were fixed 30 minutes or 50 minutes in 4% paraformaldehyde (PH 7.4), embedded in paraffin, and serially sectioned at 10 μm thickness. The sections were stained with Hematoxylin and Eosin (H&E) for routine histological examination with a light microscope.

Immunofluorescence staining

The immunofluorescence staining procedure for transfected hESC-CMs plated on Matrigel-coated glass-bottom dishes (Nest, Wuxi, China; 801001) or heart sections was performed as previously described. Cells, or heart sections were fixed and permeabilized with 0.5% Triton X-100 in PBS for 5 min and blocked with 5% BSA in PBS for 1 h at room temperature. Then, samples were incubated overnight at 4 °C with the following antibodies diluted in 3% BSA blocking solution: (1:500, α-Actinin, Sigma-Aldrich; A7732; 1:500, Cardiac Troponin T, Proteintech, Rosemont, USA; 15513-1-AP; 1:800, NKX2.5, Cell Signaling Technology, Beverly, MA, USA; 8792S; 1:500, Brachyury, R&D Systems, Minnesota, MN, USA; AF2085), followed by secondary antibody incubation and DAPI staining. Slides were viewed under a confocal microscope (Carl Zeiss LSM880).

Lipid peroxidation assay

Disassociated hESC-CMs transfected with pri-miR-222 for 72 h or primary ventricular cardiomyocytes isolated from neonatal miR-222-KI mice (P0.5) using the Primary Cardiomyocyte Isolation Kit (Thermo Scientific, Waltham, MA, USA; 88281) were incubated in 2 μm C11-BODIPY 581/591 (Invitrogen; D3861) in DMSO at 37°C for 30 min in the dark and then subjected to FACS using a BD FACSCalibur (BD Biosciences, Franklin Lakes, NJ, USA). The data were analyzed using FlowJo.

Ferroptosis in miR-222-KI mouse embryos (E12.5) was detected by immunofluorescence staining with an antibody against 4-hydroxynonenal (Abcam, Cambridgeshire, UK; ab48506)

following the same method described above.

Reverse transcription quantitative PCR (RT-qPCR) and miRNA microarray

Tissue samples were immediately snap-frozen in liquid nitrogen and stored in liquid nitrogen. Total RNA, including miRNA, from tissue samples or cultured cells was extracted using a miRNeasy Mini Kit (Qiagen, Hilden, Germany; 217004). Mature miRNA reverse transcription reactions were performed using a miRNA First-Strand cDNA Synthesis Kit (GeneCopoeia, Shanghai, China; QP014). pre-miRNA and pri-miRNA were converted to cDNA using a miScript II RT Kit (Qiagen; 218160). mRNA was reverse transcribed into cDNA with a FastQuant RT Kit with gDNase (Tiangen, Beijing, China; KR106-02). The levels of mature miRNA, pre-miRNA, pri-miRNA, and mRNA were determined by RT-qPCR using a miRNA RT-qPCR Detection Kit (GeneCopoeia; QP016), a miScript SYBR Green PCR Kit (Qiagen; 218073), TaqMan Universal Master Mix II with UNG (Applied Biosystems, Foster City, CA, USA; 4440038), and SuperReal PreMix Plus SYBR Green (Tiangen; FP205), respectively, following the manufacturers' instructions. RT-qPCR was performed using the Applied Biosystems QuantStudio 3 Real-Time PCR System in a volume of 20 μL, and *U6* or *GAPDH* expression levels were used as internal controls for normalization. The relative mature miRNA, pri-miRNA, pre-miRNA or mRNA expression levels were calculated using the $2^{-\Delta\Delta Ct}$ method.

The Affymetrix GeneChip miRNA 2.0 Array (Affymetrix, Santa Clara, CA, USA) containing 1105 human miRNA probes was used for the analysis of miRNA expression according to the manufacturer's protocols.

Antibodies

Primary antibodies against the following proteins were used in this study: α-Actinin (Sigma-Aldrich; A7732), Cardiac Troponin T (Proteintech, Rosemont, USA; 15513-1-AP), NKX2.5 (Cell Signaling Technology, Beverly, MA, USA; 8792S), Brachyury (R&D Systems, Minnesota, MN, USA; AF2085), AGO2 (Sigma-Aldrich; SAB4200085), Dicer (Abcam; ab14601), Flag Tag, (Proteintech; 66008-3), Myc-Tag, (Abmart, Shanghai, China; M20002L), and GAPDH (Proteintech; 60004-1).

The secondary antibodies were goat anti-rabbit Alexa Fluor 488 (Invitrogen; A-11008), goat

anti-mouse Alexa Fluor 594 (Invitrogen; A-11005), donkey anti-goat Alexa Fluor Plus 594 (Invitrogen; A32758), goat anti-mouse HRP (Abmart, Berkeley Heights, NJ, USA; M21001L), goat anti-rabbit HRP (Proteintech; SA00001-2), and goat anti-rat HRP (Yeasen, Wuhan, China; 33301ES60).

Plasmids

For the luciferase reporter plasmids psiCHECK2-AGO2-3′UTR and psiCHECK2-DICER1-3′UTR, a 336 bp fragment of the AGO2 3′UTR and a 524 bp fragment of the DICER1 3′UTR were amplified from human genomic DNA using the following primer sequences: forward, 5’-CCGCTCGAGTGAAAGTTGATTTGGGAGGCT -3’, reverse, 5’- ATTTGCGGCCGCCCTTCGA GCCAGATATTGCAG -3’ (AGO2-3′UTR, 336 bp); forward, 5’- CCGCTCGAGGTTCCTGTGAT TGCAATGTGAG -3’, reverse, 5’- ATTTGCGGCCGCACAGGAGTGTTAGAGGTCATTC -3’ (DICER1-3′UTR , 524 bp) by PCR and cloned into the XhoI and NotI sites of psiCHECK-2 vector (Promega, Madison, WI, USA) downstream of the Renilla luciferase gene sequence. The corresponding miRNA binding sites for miR-222 in the plasmids were mutated in the psiCHECK2-3′UTR by site-directed mutagenesis using the following primer sequences: forward, 5’-GCTTTTACacagctcATTTTGTTTTGCTTTTGTTATTTTTGT-3’, reverse, 5’- ATgagctgtGTAAAA GCTAAAGAAAGAAAATGCAGC -3’ (AGO2-3′UTR-MUT); forward, 5’- CTGTTCactgcttaCAG ATAAGCATTGCACTTGGTACC -3’, reverse, 5’- TCTGtaagcagtGAACAGAACAGAAGGGAA AAAGCT -3’ (DICER1-3′UTR-MUT) via PCR and verified by DNA sequencing and did not contain any other sequence variations.

For the lentiviral vector pLJM1-pri-miR-222, a 752 bp human genomic DNA fragment containing pri-miR-222 was amplified using the following primer sequences: forward, 5’-CTAGCTAGCCCCAAGTTAAAGTGACAGGAAG -3’, reverse, 5’- CCGGAATTCCCCAGCTG ATAATGTTGGAC -3’ (AGO2-3′UTR, 336 bp) by PCR and cloned into the NheI and EcoRI sites of pLJM1- (Addgene, Watertown, MA, USA).

For the expression plasmids AGO2 and DICER1, a 2580 bp cDNA of AGO2 and a 5769 bp DICER1 using the following primer sequences: forward, 5’- ATGTACTCGGGAGCCGGC-3’, reverse, 5’- GTAAGCAAAGTACATGGTGCGC -3’ (AGO2); forward, 5’- ATGAAAAGCCCTGC TTTGCA -3’, reverse, 5’- GTAGCTATTGGGAACCTGAGGTTG -3’ (DICER1) by PCR were

cloned into pCMV6-AC-myc and pCMV6-AC-DDK, respectively.

Pri-miR-222-expressing lentivirus

Human embryonic kidney (HEK) 293T cells were cultured on 100-mm plates at 80% confluence. 12 μg of pLJM1-pri-miR-222 and the packaging plasmids 7.8 μg of pMDL, 6 μg of pREV, and 4.2 μg of pVSVG (Addgene) were cotransfected into HEK293T cells with Lipofectamine 2000 (Invitrogen; 11668019). After forty-eight hours, the viral supernatant was collected, further concentrated by PEG 8000, and stored at -80°C. hESCs infected with the pri-miR-222-expressing lentivirus were selected with puromycin (0.2 μg/mL) for 2 weeks. The expression of miR-222 was confirmed by RT-qPCR. The hESC-CMs differentiated from hESCs infected with the pri-miR-222-expressing lentivirus are referred to as pri-miR-222-expressing hESC-CMs.

Immunoblot analysis

Cultured cells were washed in cold PBS and lysed in cold Western lysis buffer (Beyotime, Shanghai, China; p0013) with a protease inhibitor cocktail from Roche (Basel, Switzerland). Total protein from whole-cell lysate was used for immunoblot analysis by following a standard procedure. GAPDH was used as an internal control.

Luciferase reporter assay

The luciferase reporter plasmids psi-CHECK2-AGO2-3′UTR and psi-CHECK2-DICER1-3′UTR or their mutants were cotransfected into HEK293T or H9C2 cells seeded in 24-well plates with 100 nM miR-222 mimic or miR-NC mimic (GenePharma; B02004) and Lipofectamine 3000 (Invitrogen; L3000015). After thirty-six hours, the cells were washed three times using cold PBS and lysed in passive lysis buffer. Luciferase activity was measured using a Dual-Luciferase Assay System (Promega; E1960) on a GloMax-Multi Detection System plate reader (Promega).

miRNA pulldown assay

Biotin-labeled double-stranded miR-222 mimic or miR-NC mimic (GenePharma; A04004) was transfected into hESC-CMs with Lipofectamine RNAiMAX Transfection Reagent (Invitrogen; 13778075). Twenty-four hours after transfection, cells were harvested using lysis buffer with

RiboLock RNase Inhibitor (Thermo Scientific; EO0382) and a protease inhibitor cocktail (Roche; 4693116001), and RNP complexes with the target mRNAs were pulled down by Dynabeads M-280 Streptavidin (Invitrogen; 11205D). The target mRNAs were analyzed by RT-qPCR, and the enrichment of the target mRNAs was calculated as follows: fold enrichment = (AGO2 or DICER1 mRNA pulled down by miR-222/AGO2 or DICER1 mRNA pulled down by miR-NC mimic)/(Biotin-miR-222 input/Biotin-miR-NC mimic input). At least three independent experiments with three replicates were performed for each set of experiments.

Statistical Analyses

Data are presented as means ± S.D. P-values were calculated by two-sided Student's t-tests (*$p < 0.05$, ** $p < 0.01$, *** $p < 0.001$, **** $p < 0.0001$).

Extended results and discussion

MiR-222 regulates cardiomyocyte differentiation

To determine whether landmarks in the course of heart development are affected by high miR-222 expression, we generated an hESC line stably overexpressing exogenous miR-222 (Fig.S1b), while maintaining normal expression of the stem cell marker *SOX2* (Fig.S1c). The numbers of cells with positive *brachyury* (a mesoderm marker) and *NKX2-5* (a c.ardiac progenitor marker) expression were significantly elevated on day 1 (Fig.S1e) and day 5 (Fig.S1f) during hESC-CMs differentiation among the cells with stable miR-222-overexpression (Fig.S1d). Moreover, from an early stage of cardiomyocyte differentiation, many downstream or TOF-related genes with functions in stem cell pluripotency, mesoderm, cardiac mesoderm, cardiac progenitor cells, and immature cardiomyocytes were altered with miR-222 overexpression (Fig.S1g). Taken together, these findings suggest that abnormally high miR-222 expression levels in the hearts contribute to the pathogenesis of TOF from an early stage of heart development.

DICER1 regulates the maturation of miR-222 and constitutes a feedback loop

The level of mature miR-222 expression was decreased in hESC-CMs treated with siRNA

against *DICER1* (Fig. S3j, k), confirming the role of *DICER1* in the biogenesis of miR-222. The observations that miR-222 biogenesis depends on *DICER1* and the upregulation of miR-222 inhibits *DICER1* expression suggested that a feedback system may exist to maintain a low level of miR-222 in a physiological state.

MiR-222 induces DICER1/AGO2-dependent ferroptosis via *FTH1* and *NOX1*

Ferroptosis can be triggered by an iron overload and the dysregulation of ROS metabolism[3]. The knockdown of *DICER1* or *AGO2* also resulted in increased lipid peroxide levels (Fig. S5c, S5d). *FTH1* expression is negatively related to intracellular iron storage, and *NOX1* stimulates the production of superoxide and hydrogen peroxide[3]. Mechanistically, the downregulation of *DICER1* expression reduced the expression of *FTH1* (Fig.S5f) and subsequently led to the accumulation of iron, while the downregulation of *AGO2* expression increased the expression of *NOX1* (Fig.S5g) and consequently promoted peroxidation overload. These results suggest that the upregulation of miR-222 expression can induce DICER1/AGO2-dependent ferroptosis, which may affect TOF development.

Discussion

The processing of canonical miRNAs from primary miRNAs (pri-miRNAs) to precursor miRNAs (pre-miRNAs) to mature miRNAs involves multiple steps, and *DICER1* and *AGO2* are mainly responsible for the processing of pre-miRNAs to mature miRNAs[4]. Interference with *DICER1* or *AGO2* markedly reduces mature miRNA levels [4,5]. There have been reported that the deficiency of *Dicer1* and *Ago2* lead to cardiac defect in mouse model. Embryos lacking *Dicer* in the developing heart exhibited pericardial edema and a poorly developed ventricular myocardium, and the embryos died from cardiac failure by E12.5[6]. *Ago2*-deficient mice displayed pericardial edema and enlarged heart, and suffer from cardiac failure[7,8]. In this study, we proved that *DICER1* and *AGO2* participated in the developing of TOF via the regulation of miRNA maturation and ferroptosis both *in vitro* and *in vivo*, which further highlight their vital role in cardiac development.

Approximately 100 differentially expressed microRNAs have been identified in TOF[9-13]. However, no individual miRNA has been reported to cause TOF phenotypes in an animal model. In the present study, we found that miR-222 expression was dramatically upregulated in the hearts of

human fetuses with TOF and that the changes in mir-222 KI mice phenocopied those observed in human TOF. MiR-222 participates in a variety of physiological and pathological processes in the heart[14]. MiR-222 induces cardiomyocyte proliferation and hypertrophy *in vitro*; however, its function *in vivo* in the heart is controversial. In particular, the constitutive expression of mmu-mir-222 in the heart after birth leads to heart failure in adult mice[15], whereas inducible expression of mmu-mir-222 in the adult mouse heart protects against adverse ventricular remodeling and cardiac dysfunction after ischemic injury[16,17]. However, the role of miR-222 in the developing heart remains unclear. Our results revealed that exogenous miR-222 simultaneously downregulates the expression of *DICER1* and *AGO2*. Consequently, miR-222-*DICER1-AGO2* signaling inhibits cardiac miRNA maturation, decreases cardiac miRNA dosage, and induces ferroptosis, which may eventually contribute to TOF. Together, our findings clarify the vital roles of the master regulator miR-222 in TOF development and its underlying mechanisms of action.

To date, seven human TOF-associated genes have been individually mutated in mice, including *Gata4*, *Gata6*, *Fog2/Zfpm2*, *Jag1*, *Nkx2-5*, *Tbx1,* and *Tbx5*[18-22]. *Jag1*-deficient mice have been developed as a model of TOF[23], and none of the other homozygous mutants effectively recapitulate human TOF. Although two TOF-associated genes, *Gata4* and *Fog2/Zfpm2,* were predicted as targets of miR-222, they were not regulated by miR-222 in further experiments for validation. Our mir-222 KI mouse model in which the β-MHC promoter was used to drive exogenous mir-222 expression in cardiomyocytes from E9.5[24] was different from the previously reported Tg-mir-222 mouse model using α-MHC[15], a cardiac promoter functioning after birth. Accordingly, the previous mouse model did not display any TOF phenotypes, while our new mouse model recapitulated the typical defects of human TOF, providing the first mouse model of TOF through miRNA manipulation.

Mechanistically, we revealed that miR-222 targets both *DICER1* and *AGO2*, which work together to achieve the maturation of most miRNAs. Only a few miRNAs are known to engage in negative feedback with either *DICER1* or *AGO2* separately, as observed in cancers[25-28] and erythroid cells[29]. Here, we demonstrate that miR-222 is thus far unique miRNA, which simultaneously targets both *DICER1* and *AGO2* in a feedback loop. As a dual regulator, we propose that miR-222 blocks the functional redundancy of *DICER1* and *AGO2* and has an additive effect on heart development. This is supported by the observation that exogenous miR-222 substantially reduced the levels of mature cardiac-related miRNAs by as much as 90–95% *in vitro* and in the hearts of our mir-222-KI

mice. In contrast, miRNA maturation was decreased only 30–70% by let-7[25] and miR-376a[29], which negatively target *DICER1* and *AGO2*, respectively.

In heart development, caspase-dependent apoptosis almost certainly contributes to developmental vessel regression[30]. However, it is not clear whether caspase-independent cell death pathways are involved in heart development or in the differentiation of CMs. Here, we demonstrated that ferroptosis was elevated in the hearts of fetuses with TOF and in CMs from the heart tissues of mir-222-KI mice at E12.5 and P0.5. Ferroptosis is also involved in ischemia-induced cardiomyopathy in mice[31] and is increased in heart tissues of patients with RV failure[32]. Based on these reported data, we speculate that ferroptosis is the form of cell death that sustains the homeostasis of CMs under stress conditions, such as abnormally high miR-222 levels, and might be a pathogenic factor in TOF, which deserves further study.

In summary, we identified miR-222 as a master regulator of cardiac miRNA maturation/dosage and demonstrated connections among high levels of miR-222, cardiac hypertrophy, and ferroptosis in mouse models and fetuses with TOF. Importantly, our *in vivo* data showed that miR-222-mediated hypertrophy, ferroptosis, and other TOF-featured cardiac defects could be significantly rescued by treatment with a miRNA maturation enhancer or ferroptosis inhibitor. Therefore, our research shed light on the role of miR-222-*DICER1*-*AGO2* signaling in the control of cardiac miRNA maturation/dosage and ferroptosis as potential therapeutic targets for the prevention of TOF. It would be interesting to determine whether treatment with enoxacin or a ferroptosis inhibitor is effective in different congenital heart disease models and in patients with TOF.

Reference

1. Rindt, H., Gulick, J., Knotts, S., Neumann, J. & Robbins, J. In vivo analysis of the murine beta-myosin heavy chain gene promoter. *J. Biol. Chem.* **268**, 5332-5338 (1993).
2. Lian, X. et al. Robust cardiomyocyte differentiation from human pluripotent stem cells via temporal modulation of canonical Wnt signaling. *Proc. Natl. Acad. Sci. U. S. A.* **109**, E1848-1857 (2012).
3. Xie, Y. et al. Ferroptosis: process and function. *Cell Death Differ.* **23**, 369-379 (2016).
4. Kim, Y. K., Kim, B. & Kim, V. N. Re-evaluation of the roles of DROSHA, Export in 5, and DICER in microRNA biogenesis. *Proc. Natl. Acad. Sci. U. S. A.* **113**, E1881-1889 (2016).
5. Golden, R. J. et al. An Argonaute phosphorylation cycle promotes microRNA-mediated silencing. *Nature* **542**, 197-202 (2017).
6. Zhao, Y. et al. Dysregulation of cardiogenesis, cardiac conduction, and cell cycle in mice lacking miRNA-1-2. *Cell* **129**, 303-317 (2007).
7. Liu, J. et al. Argonaute2 is the catalytic engine of mammalian RNAi. *Science* **305**, 1437-1441 (2004).
8. Cheloufi, S., Dos Santos, C. O., Chong, M. M. & Hannon, G. J. A dicer-independent miRNA biogenesis pathway that requires Ago catalysis. *Nature* **465**, 584-589 (2010).
9. O'Brien, J. E., Jr. et al. Noncoding RNA expression in myocardium from infants with tetralogy of Fallot. *Circ. Cardiovasc. Genet.* **5**, 279-286 (2012).
10. Wang, B., Shi, G., Zhu, Z., Chen, H. & Fu, Q. Sexual difference of small RNA expression in Tetralogy of Fallot. *Sci. Rep.* **8**, 12847 (2018).
11. Wu, Y. et al. Expression of Cx43-related microRNAs in patients with tetralogy of Fallot. *World J. Pediatr.* **10**, 138-144 (2014).
12. Zhang, J. et al. MicroRNA deregulation in right ventricular outflow tract myocardium in nonsyndromic tetralogy of fallot. *Can. J. Cardiol.* **29**, 1695-1703 (2013).
13. Liang, D. et al. miRNA-940 reduction contributes to human Tetralogy of Fallot development. *J. Cell. Mol. Med.* **18**, 1830-1839 (2014).
14. Ding, S., Huang, H., Xu, Y., Zhu, H. & Zhong, C. MiR-222 in Cardiovascular Diseases: Physiology and Pathology. *Biomed Res Int* **2017**, 4962426 (2017).
15. Su, M. et al. Cardiac-Specific Overexpression of miR-222 Induces Heart Failure and Inhibits Autophagy in Mice. *Cell. Physiol. Biochem.* **39**, 1503-1511 (2016).
16. Vujic, A. et al. Exercise induces new cardiomyocyte generation in the adult mammalian heart. *Nat Commun* **9**, 1659 (2018).
17. Liu, X. et al. miR-222 is necessary for exercise-induced cardiac growth and protects against pathological cardiac remodeling. *Cell Metab.* **21**, 584-595 (2015).
18. Di Felice, V. & Zummo, G. Tetralogy of fallot as a model to study cardiac progenitor cell migration and differentiation during heart development. *Trends Cardiovasc. Med.* **19**, 130-135 (2009).
19. Zhang, W. et al. GATA4 mutations in 486 Chinese patients with congenital heart disease. *Eur. J. Med. Genet.* **51**, 527-535 (2008).
20. Lin, X. et al. A novel GATA6 mutation in patients with tetralogy of Fallot or atrial septal defect. *J. Hum. Genet.* **55**, 662-667 (2010).
21. Rauch, R. et al. Comprehensive genotype–phenotype analysis in 230 patients with tetralogy of Fallot. *J. Med. Genet.* **47**, 321-331 (2010).

22. Baban, A. et al. Identification of TBX5 mutations in a series of 94 patients with Tetralogy of Fallot. *Am. J. Med. Genet. A* **164**, 3100-3107 (2014).
23. Hofmann, J. J. et al. Endothelial deletion of murine Jag1 leads to valve calcification and congenital heart defects associated with Alagille syndrome. *Development* **139**, 4449-4460 (2012).
24. Luo, Y. et al. Rescuing the N-cadherin knockout by cardiac-specific expression of N-or E-cadherin. *Development* **128**, 459-469 (2001).
25. Tokumaru, S., Suzuki, M., Yamada, H., Nagino, M. & Takahashi, T. let-7 regulates Dicer expression and constitutes a negative feedback loop. *Carcinogenesis* **29**, 2073-2077 (2008).
26. Cochrane, D. R. et al. MicroRNAs link estrogen receptor alpha status and Dicer levels in breast cancer. *Horm. Cancer* **1**, 306-319 (2010).
27. Martello, G. et al. A MicroRNA targeting dicer for metastasis control. *Cell* **141**, 1195-1207 (2010).
28. Kovaleva, V. et al. miRNA-130a targets ATG2B and DICER1 to inhibit autophagy and trigger killing of chronic lymphocytic leukemia cells. *Cancer Res.* **72**, 1763-1772 (2012).
29. Wang, F., Yu, J., Yang, G. H., Wang, X. S. & Zhang, J. W. Regulation of erythroid differentiation by miR-376a and its targets. *Cell Res.* **21**, 1196-1209 (2011).
30. Fisher, S. A., Langille, B. L. & Srivastava, D. Apoptosis during cardiovascular development. *Circul. Res.* **87**, 856-864 (2000).
31. Fang, X. et al. Ferroptosis as a target for protection against cardiomyopathy. *Proc. Natl. Acad. Sci. U. S. A.* **116**, 2672-2680 (2019).
32. Hwang, H. V. et al. 4HNE Impairs Myocardial Bioenergetics in Congenital Heart Disease-Induced Right Ventricular Failure. *Circulation* **142**, 1667-1683 (2020).

Supplemental Figures

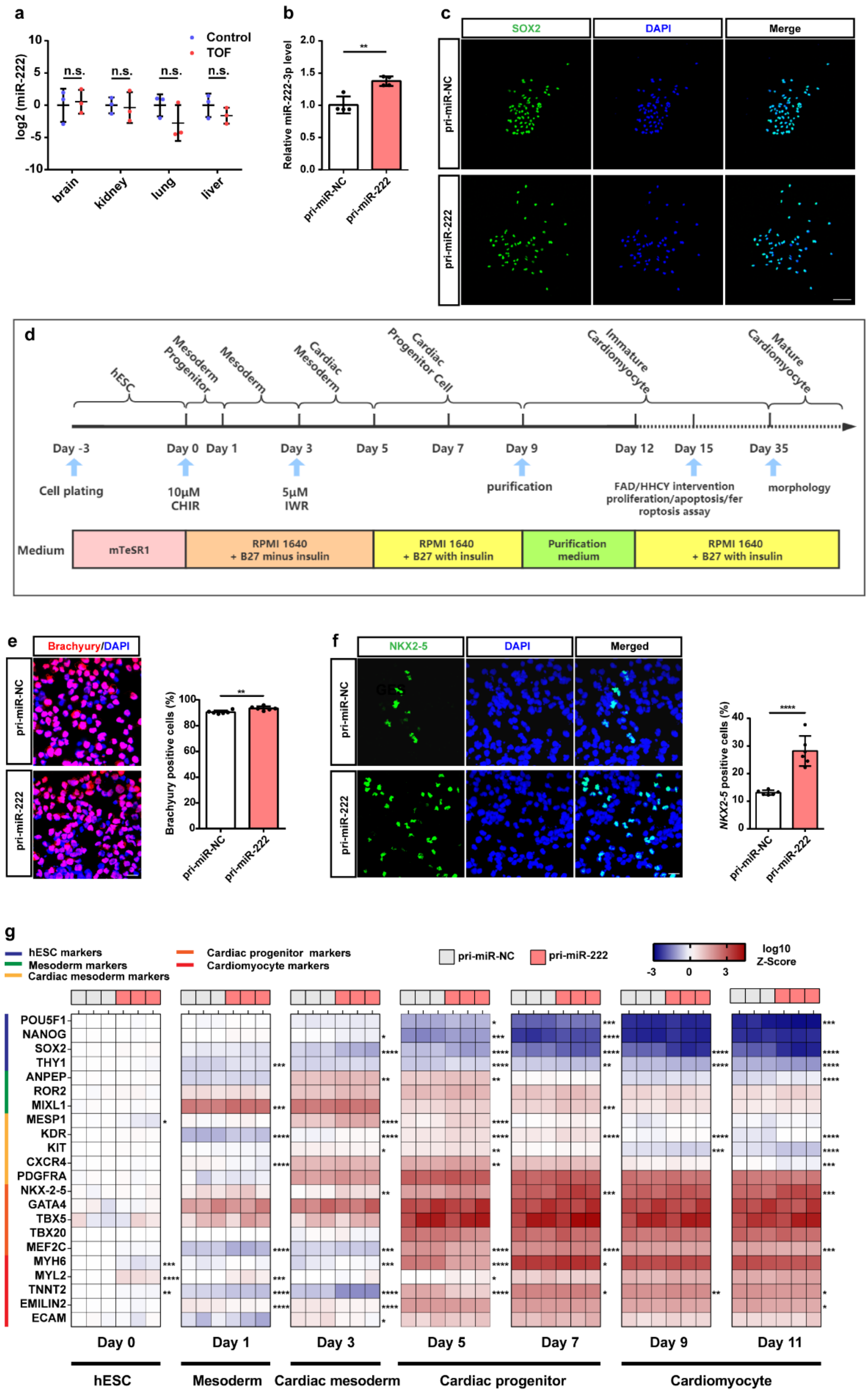

Figure. S1. MiR-222 promotes cardiomyocyte differentiation a, RT-qPCR analysis of miR-222 levels in the brains, kidneys, livers and lungs (n=3) of aborted fetuses with TOF and normal controls. **b, c,** RT-qPCR analysis of miR-222 level (**b**) and SOX2-immunofluorescence staining for pluripotency marker SOX2 (green) (**c**) in hESCs infected with lenti-pri-miR-222 or control virus (n=4). Scale bars in (**c**) are 100 μm. **d,** Schematic of protocol for differentiation from hESCs to cardiomyocytes though treatment with small molecules. **e, f,** Immunofluorescence staining of the mesoderm marker *Brachyury* (**e**) and the cardiac progenitor marker NKX2-5 (**f**) in pri-miR-222-expressing hESC-CMs and control cells and quantification of Brachyury-positive and NKX2-5-positive hESC-CMs. At least six randomly selected fields were counted for each group, and the experiment was repeated three times. The number above each bar indicates the total number of cells counted. **g**, RT-qPCR analyses of expression levels of various markers for stem cell pluripotency, mesoderm, cardiac mesoderm, cardiac progenitor cells, and cardiomyocytes during differentiation from hESCs to cardiomyocytes (n = 4). Nuclei are stained with DAPI in (**e**) and (**f**). At least 100 cells in (**e**) and (**f**) were quantified in each group. The scale bars in (**e**) and (**f**) are 20 μm. Data are shown as means ± S.D. *$p < 0.05$, ** $p < 0.01$, *** $p < 0.001$, **** $p < 0.0001$

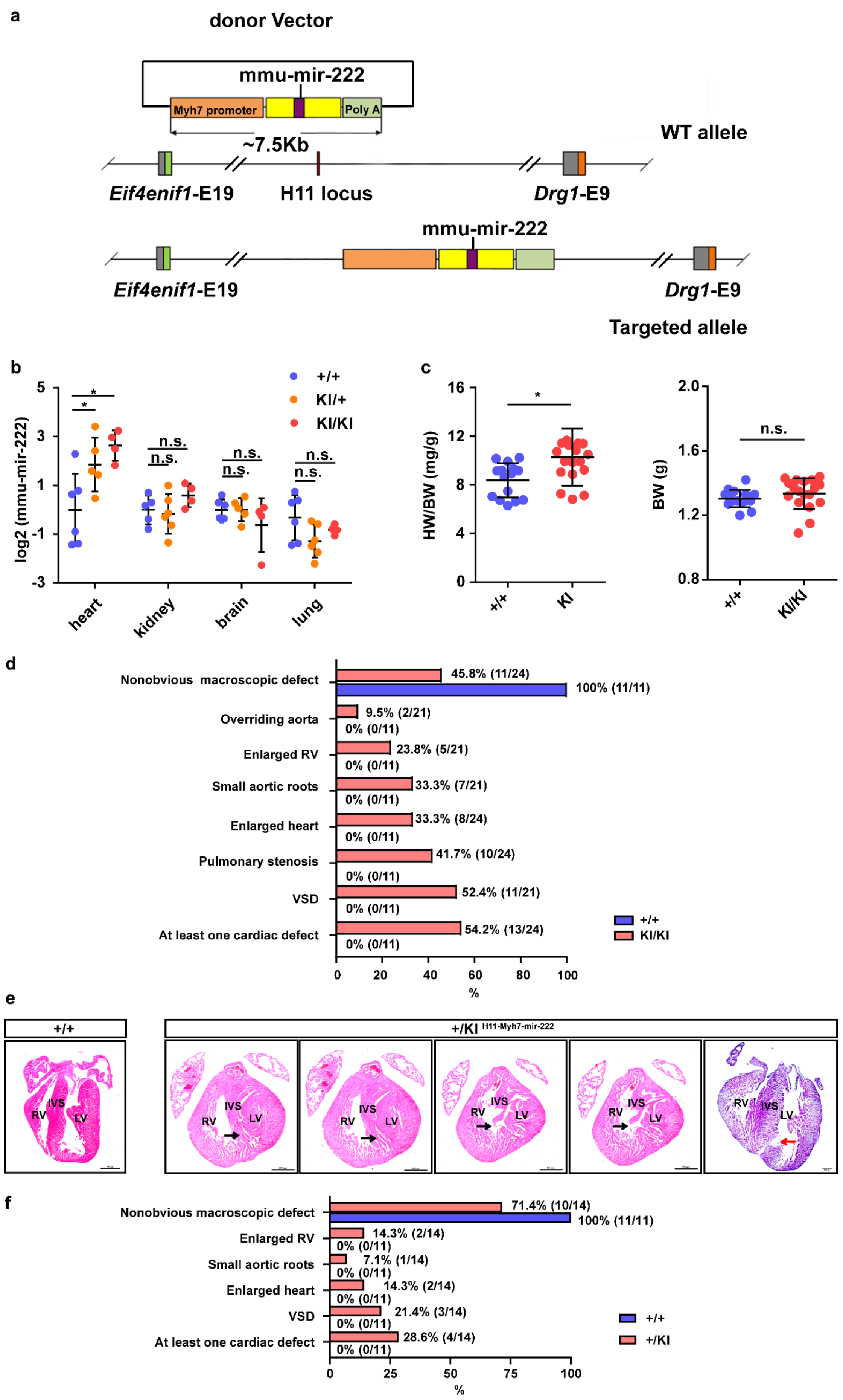
a
donor Vector
mmu-mir-222
Myh7 promoter
Poly A
~7.5Kb
WT allele
Eif4enif1-E19
H11 locus
Drg1-E9
mmu-mir-222
Eif4enif1-E19
Drg1-E9
Targeted allele
b
log2 (mmu-mir-222)
+/+
KI/+
KI/KI
n.s.
heart
kidney
brain
lung
c
HW/BW (mg/g)
BW (g)
+/+
KI
KI/KI
n.s.
d
Nonobvious macroscopic defect
45.8% (11/24)
100% (11/11)
Overriding aorta
9.5% (2/21)
0% (0/11)
Enlarged RV
23.8% (5/21)
0% (0/11)
Small aortic roots
33.3% (7/21)
0% (0/11)
Enlarged heart
33.3% (8/24)
0% (0/11)
Pulmonary stenosis
41.7% (10/24)
0% (0/11)
VSD
52.4% (11/21)
0% (0/11)
At least one cardiac defect
54.2% (13/24)
0% (0/11)
+/+
KI/KI
%
e
+/+
+/KI H11-Myh7-mir-222
RV
IVS
LV
f
Nonobvious macroscopic defect
71.4% (10/14)
100% (11/11)
Enlarged RV
14.3% (2/14)
0% (0/11)
Small aortic roots
7.1% (1/14)
0% (0/11)
Enlarged heart
14.3% (2/14)
0% (0/11)
VSD
21.4% (3/14)
0% (0/11)
At least one cardiac defect
28.6% (4/14)
0% (0/11)
+/+
+/KI
%

Figure. S2. Cardiac-specific expression of exogenous miR-222 drives TOF-related phenotypes

a, Schematic illustration of strategy for knocking in β-MHC-mir-222 at the H11 locus. **b,** RT-qPCR analysis of mir-222 levels in the hearts, kidneys, brains, and lungs of P0.5 neonatal mice with the indicated genotypes. **c,** Heart weight/body weight ratio (**left**) and body weight (**right**) of P0.5 neonatal homozygous mir-222-knock-in (KI) and control mice. **d,** Quantification of cardiac defect number according to stereoscopic images and H&E-stained sections of whole hearts of homozygous mir-222 KI and control mice. (3 of the 24 pups' hearts were isolated to process with FACS)**. e,** H&E-stained heart sections from heterozygous mir-222 KI and control mice, displaying human TOF-like phenotypes, A mir-222 KI/+ littermate of the animal in (**right**) shows RV hypertrophy , VSDs (arrow) at P0.5. The scale bars in (**left**) and (**right**) are 500 μm and 200 μm, respectively. **f**, Quantification of cardiac defect number according to H&E-stained sections of whole hearts of heterozygous mir-222 KI and control mice.

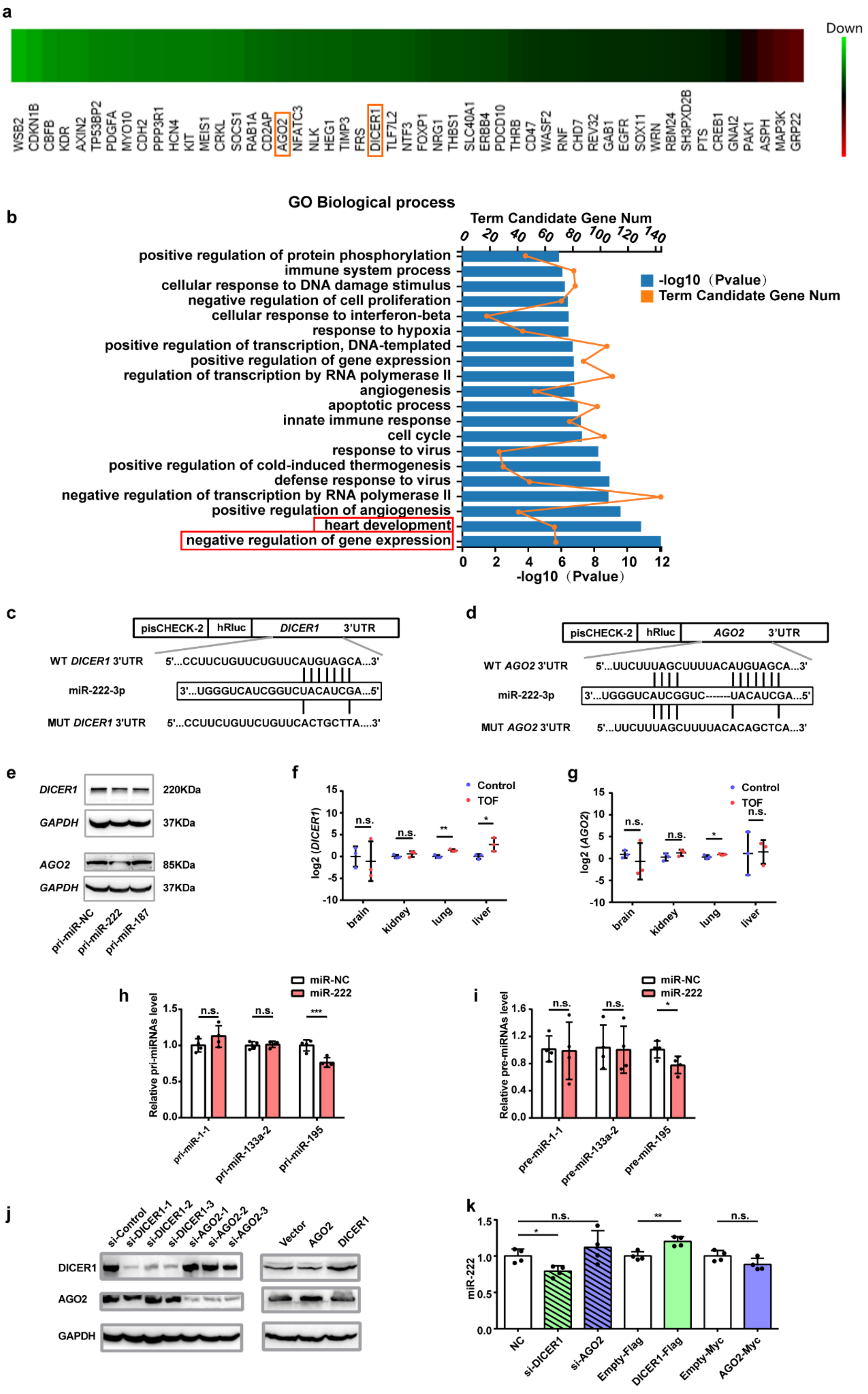

Figure. S3. MiR-222 disturbs miRNA maturation a, The heat map of RT-qPCR results showing

the expression levels of candidate target genes of miR-222 in hESC-CMs transfected with miR-222. **b,** Gene Ontology analysis of differentially regulated genes in KI/KI hearts at P0.5. **c**, **d**, Schematic illustration of luciferase reporters containing WT and mutant miR-222 binding sites in the *DICER1* 3′UTR (**c**) and *AGO2* 3′UTR (**d**).**e,** Western blot analysis of AGO2 and DICER1 in hESCs infected by lenti-pri-miR-222, lenti-pri-miR-187 or scramble control lentivirus. **f, g,** RT-qPCR analyses of levels of *DICER1* (**f**) and *AGO2* (**g**) in brains, kidneys, livers and lungs of aborted fetuses with TOF (n=3) and normal controls (n=3). **h, i,** RT-qPCR analysis of the levels of and pri- (**h**) or pre- (**i**) miR-1, miR-133a and miR-195 in hESC-CMs transfected with miR-222 or scramble control for 72 h. The experiments were repeated four times.**j,** Western blotting of AGO2 and DICER1 in hESC-CMs transfected with siRNAs (left panel) or *AGO2* or *DICER1* expressed vector (right panel) as indicated. GAPDH was used as a loading control. **k,** RT-qPCR analyses of levels of miR-222 in hESC-CMs transfected with si-*DICER1*, si-*AGO2* or scramble control and *DICER1* or *AGO2*-expression vectors (n=4). GAPDH (**e-i**) was used as internal control. Data are shown as the means ± S.D. *$p < 0.05$, ** $p < 0.01$.

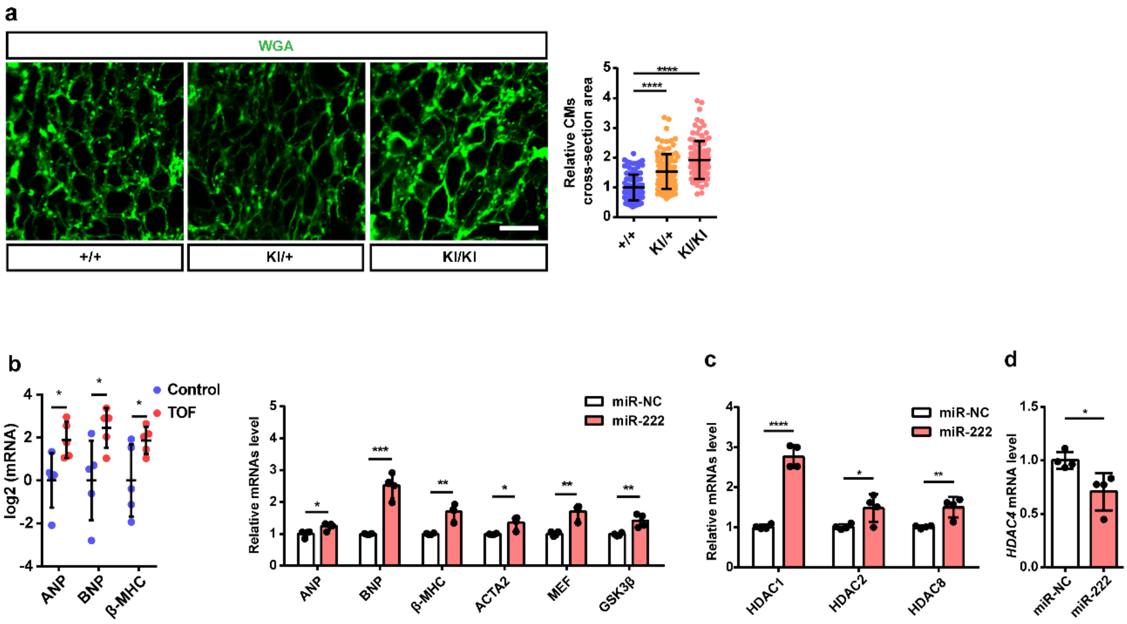

Figure. S4. MiR-222 induces cardiomyocyte hypertrophy a, Immunofluorescence staining of heart sections from E12.5 mouse embryo with WGA and quantification of cross-section area of CMs. Scale bar in (A) =10 µm. **b,** RT-qPCR analysis of a marker of cardiomyocyte hypertrophy (*ANP*, *BNP* and *β-MHC*) in RV tissues from aborted fetuses with or without TOF (n = 5 each) (**left**). RT-qPCR analyses of markers of cardiomyocyte hypertrophy in hESC-CMs transfected with miR-222 or scramble control (n = 3) (**right**). **c-d,** RT-qPCR analyses of markers of pro-hypertrophic (**c**) and anti-hypertrophic *HDAC*s (**d**) in hESC-CMs transfected with miR-222 or scramble control (n = 3). *GAPDH* was used as an internal control. Data are shown as means ± S.D. *$p < 0.05$, ** $p < 0.01$, *** $p < 0.001$, **** $p < 0.0001$

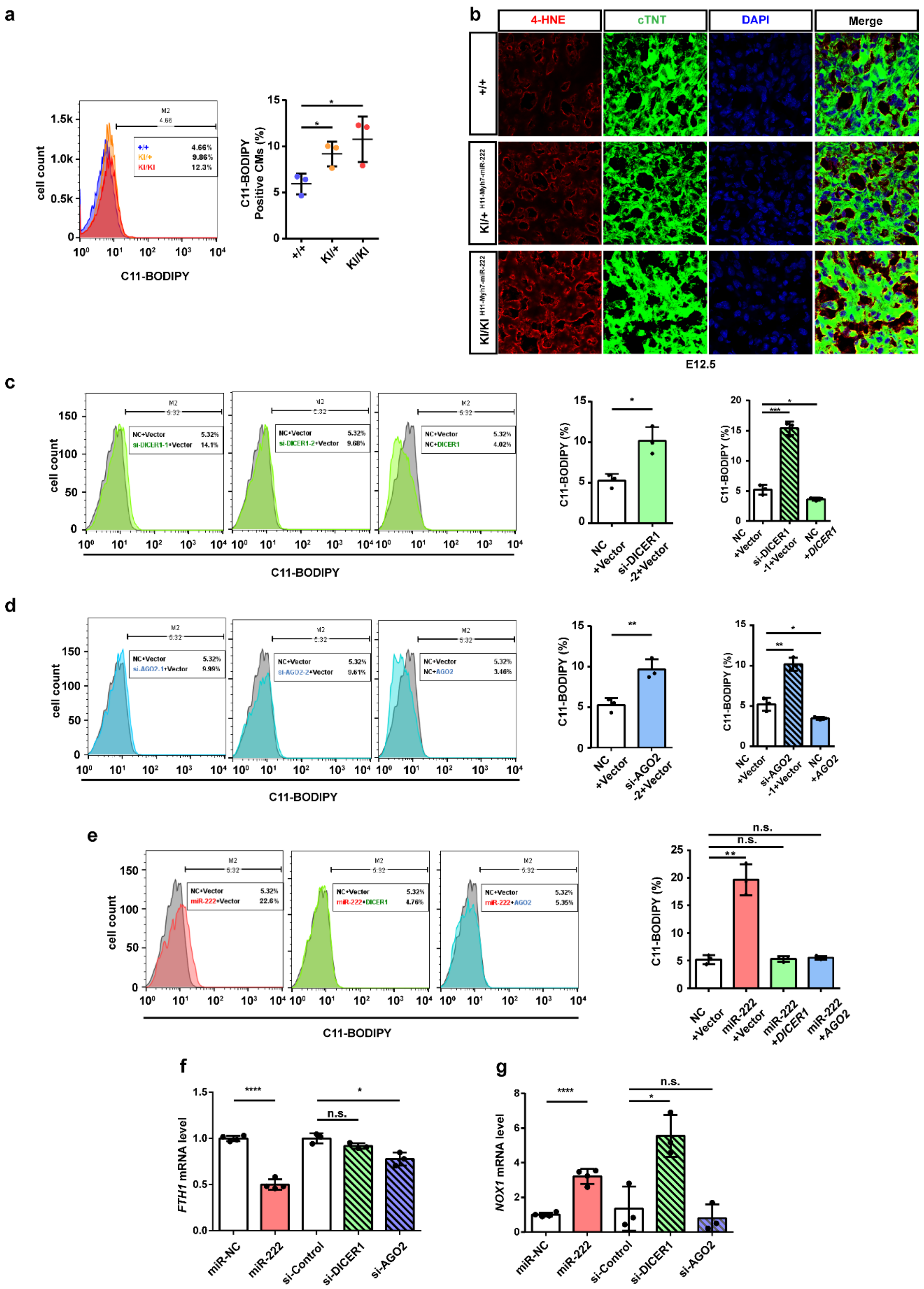

Figure. S5. MiR-222 induces ferroptosis in CMs a, FACS analysis and quantification of lipid ROS levels (C11-BODIPY) in CMs isolated from P0.5 neonatal mice with the indicated genotypes (n = 3). **b,** Representative immunofluorescence staining of cTnT and the lipid ROS marker 4-HNE in heart sections from E12.5 with the indicated genotypes **c, d,** FACS analysis and quantification of

lipid ROS levels in hESC-CMs transfected with *DICER1* or *AGO2*-expression vectors or knockdown of *DICER1* or *AGO2*. **e,** FACS analysis and quantification of lipid ROS levels in hESC-CMs cotransfected with miR-222 and *AGO2* or *DICER1* expression vectors by Lipofectamine 3000. FACS analysis and quantification of lipid ROS levels in hESC-CMs transfected with miR-222 or scramble control only (left panel) or cotransfected with *DICER1* or *AGO2* expression vectors (middle two panels). **f, g,** RT-qPCR analyses of the mRNA levels of *FTH1* (**f**) and *NOX1* (**g**) in hESC-CMs transfected with miR-222, si-*DICER1* si-*AGO2* or scramble control. *GAPDH* was used as internal control. The scale bars in (**b**) represent 10 μm. Data are shown as means ± S.D. *$p < 0.05$, ** $p < 0.01$, *** $p < 0.001$, **** $p < 0.0001$

a

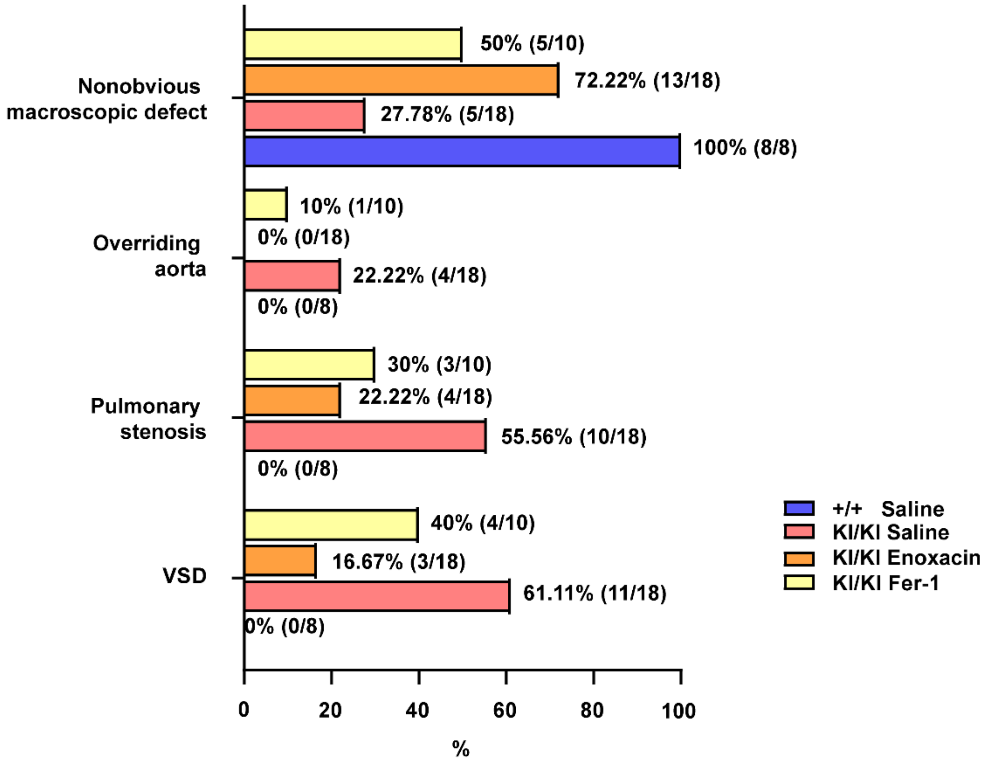

Figure. S6. miR-222-induced cardiac defects could be reversed by enoxacin or Fer-1. a, Quantification of cardiac defect number according to stereoscopic images and H&E-stained sections of whole hearts of control mice and homozygous mir-222 KI treated with saline, enoxacin, or Fer-1, as indicated.

Supplemental Tables

Table S1. Gestational age and Sex of subjects.

	n	Gender	Gestational age(Range)
TOF	5	3M/2F	23.4W(±5)
control	5	3M/2F	19.2W(±5)

Table S2. MiRNA expression in normal human heart development by microarray.

See the Supplemental Excel File I.

Table S3. Filtering with the MGI mouse phenotype database

See the Supplemental Excel File II.

Table S4. List of primer, mimic and inhibitor sequences used in this study

See the Supplemental Excel File III.
